# Performance Analysis of Latency-Aware Data Management in Industrial IoT Networks †

**DOI:** 10.3390/s18082611

**Published:** 2018-08-09

**Authors:** Theofanis P. Raptis, Andrea Passarella, Marco Conti

**Affiliations:** Institute of Informatics and Telematics, National Research Council, 56124 Pisa, Italy; andrea.passarella@iit.cnr.it (A.P.); marco.conti@iit.cnr.it (M.C.)

**Keywords:** Industry 4.0, data management, Internet of Things, performance analysis, experimental evaluation

## Abstract

Maintaining critical data access latency requirements is an important challenge of Industry 4.0. The traditional, centralized industrial networks, which transfer the data to a central network controller prior to delivery, might be incapable of meeting such strict requirements. In this paper, we exploit distributed data management to overcome this issue. Given a set of data, the set of consumer nodes and the maximum access latency that consumers can tolerate, we consider a method for identifying and selecting a limited set of proxies in the network where data needed by the consumer nodes can be cached. The method targets at balancing two requirements; data access latency within the given constraints and low numbers of selected proxies. We implement the method and evaluate its performance using a network of WSN430 IEEE 802.15.4-enabled open nodes. Additionally, we validate a simulation model and use it for performance evaluation in larger scales and more general topologies. We demonstrate that the proposed method (i) guarantees average access latency below the given threshold and (ii) outperforms traditional centralized and even distributed approaches.

## 1. Introduction

**Figure 1:**
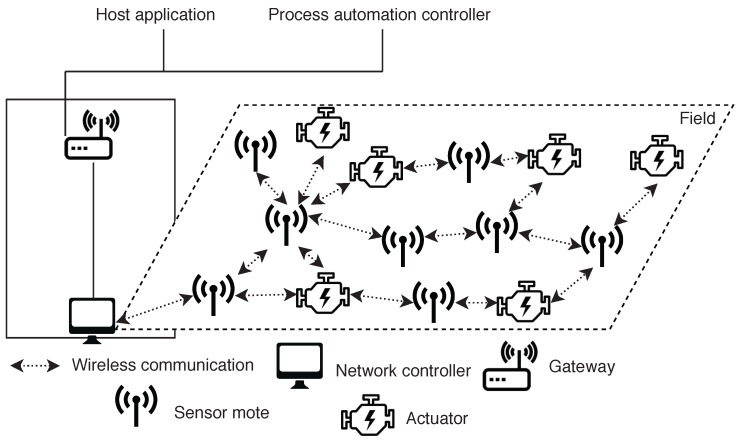
A typical industrial IoT network for condition monitoring.

Industry 4.0 refers to the fourth industrial revolution that transforms industrial manufacturing systems into Cyber-Physical Production Systems (CPPS) by introducing emerging information and communication paradigms, such as the Internet of Things (IoT) [1]. Two technological enablers of the Industry 4.0 are (i) the communication infrastructure that will support the ubiquitous connectivity of CPPS [2] and (ii) the data management schemes built upon the communication infrastructure that will enable efficient data distribution within the factories of the future [3]. Industrial IoT networks (Figure 1) are typically used, among others, for condition monitoring, manufacturing processes and predictive maintenance [4]. To maintain the stability and to control the performance, those industrial applications impose stringent end-to-end latency requirements on data communication between hundreds or thousands of network nodes [5], e.g., end-to-end latencies of 1–100 ms [6]. Missing or delaying important data may severely degrade the quality of control [7].

Edge computing, also referred to as fog computing, implements technical features that are typically associated with advanced networking and can satisfy those requirements [8]. Fog computing differs from cloud computing with respect to the actual software and hardware realizations, as well as in being located in spatial proximity to the data consumer (for example, the user could be a device in the industrial IoT case). In particular, components used to realize the fog computing architecture can be characterized by their non-functional properties. Such non-functional properties are, for example, real-time behavior, reliability and availability. Furthermore, fog nodes can follow industry-specific standards (e.g., IEEE 802.15.4e [9] or WirelessHART [10]) that demand the implementation, as well as verification and validation of software and/or hardware to follow formal rules.

Distributed data management, a key component of fog computing, can be a very suitable approach to cope with these issues [11]. In the context of industrial networks, one could leverage the set of nodes present at the edge of the network to distribute functions that are currently being implemented by a central controller [12]. Many flavors of distributed data management exist in the networking literature, depending on which edge devices are used. In this paper, we consider a rather extreme definition of distributed data management and use the multitude of sensor nodes present in an industrial physical environment (e.g., a specific factory) to implement a decentralized data distribution, whereby sensor nodes cache data they produce and provide these data to each other upon request. In this case, the choice of the sensor nodes where data are cached must be done to guarantee a maximum delivery latency to nodes requesting those data.

In this paper, we exploit the Data Management Layer (DML), which operates independently of and complements the routing process of industrial IoT networks. Assuming that applications in such networks require that there is (i) a set of producers generating data (e.g., IoT sensors), (ii) a set of consumers requiring those data in order to implement the application logic (e.g., IoT actuators) and (iii) a maximum latency $L_{max}$ that consumers can tolerate in receiving data after they have requested them, the DML offers an efficient method for regulating the data distribution among producers and consumers. The DML selectively assigns a special role to some of the network nodes, that of the proxy. Each node that can become a proxy potentially serves as an intermediary between producers and consumers, even though the node might be neither a producer, nor a consumer. If properly selected, proxy nodes can significantly reduce the access latency; however, when a node is selected as a proxy, it has to increase its storing, computational and communication activities. Thus, the DML minimizes the number of proxies, to reduce as much as possible the overall system resource consumption (the coherency of data that reside on proxies can be achieved in a variety of ways [13] and is beyond the scope of this paper). More specifically, our contributions are the following:

- We propose a distributed data management approach to store data in a number of locations in an industrial environment, as opposed to the current industrial state-of-the-art approaches where all data are centrally stored and served from a unique location. We exploit the DML for minimizing the number of proxies in an industrial IoT network and to reduce as much as possible the overall system resource consumption.
- We provide a multi-faceted performance evaluation, both through experiments and through simulations, for achieving scales much larger than what available experimental conditions allow. At first, we implement the DML with 95 real devices and evaluate its performance on the FIT IoT-LAB testbed [14]. Then, we use the simulation model, validate it against the experimental results and evaluate the DML performance in larger network sizes and more general topologies.
- We demonstrate that the proposed method (i) guarantees that the access latency stays below the given threshold and (ii) significantly outperforms traditional centralized and even distributed approaches, both in terms of average data access latency and in terms of maximum latency guarantees.
- We also demonstrate an additional flexibility of the proposed approach, by showing that it can be tuned both to guarantee that the average of the mean latency stays below $L_{\max}$ or that the average of the worst-case latency stays below $L_{\max}$ .

Roadmap of the paper: In Section 2, we provide a brief summary of concepts related to this paper. In Section 3, we provide the model of the settings we consider, as well as the necessary notation. In Section 4, we introduce the DML and the problem that it addresses. In Section 5, we evaluate the performance of the DML in comparison with two other methods used in industrial environments. We also validate the simulation model used afterwards. In Section 6, we present simulation results in scenarios that are not possible to evaluate with the available experimental testbeds. Finally, we conclude and provide insights for future work in Section 7.

## 2. Literature Review

A note on distributed data management: Traditionally, industrial application systems tend to be entirely centralized. For this reason, distributed data management has not been studied extensively in the past, and the emphasis has been put on the efficient computer communication within the industrial environment. The reader can find state-of-the-art approaches on relevant typical networks in [5,15,16]. In [17], we defined the concept of a data management layer separate from routing functions for industrial wireless networks. The DML considered in the current paper is similar to the one defined in [17], although here, we make it more practical. More specifically, in [17], we focused on a graph-theoretic approach, which might sometimes be unrealistic when real industrial implementations with technological constraints are at hand. Furthermore, in [17], we did not provide an extensive experimental evaluation of our methods, as is done in the current paper, but we verified the methods solely via simulations. Even more importantly, in this paper, we provide (i) a real implementation of the DML, (ii) experimental results on its performance, (iii) a validation of a DML simulation model and (iv) a large-scale performance evaluation of the DML using the validated simulation model.

Related works: We now provide some additional interesting related works. In [18], although the authors considered delay and real-time aspects, the main optimization objectives were the energy efficiency and reliability. They presented a centralized routing method, and consequently, they did not use proxies. Furthermore, the model of this paper assumes that the network is operating under different protocols (e.g., 802.11). In [19], the authors addressed a different optimization objective, focusing on minimizing the maximum hop distance, rather than guaranteeing it as a hard constraint. Furthermore, they assumed a bounded number of proxies, and they examined only on the worst-case number of hops. Finally, the presented approach was somewhat graph-theoretic, which made it hard to apply to real industrial IoT networks. In [20], the authors, given the operational parameters required by the industrial applications, provided several algorithmic functions that locally reconfigured the data distribution paths, when a communication link or a network node failed. They avoided continuously recomputing the configuration centrally, by designing an energy-efficient local and distributed path reconfiguration method. However, due to the locality of the computations and the complete absence of a central coordination, this method may result in violating the latency requirements. In [21], the authors presented a cross-layer approach, which combined MAC-layer and cache management techniques for adaptive cache invalidation, cache replacement and cache prefetching. Again, the model is different, as we assume a completely industrially-oriented MAC layer, based on IEEE802.15.4e, and a different problem, focusing on the delay aspects, instead of cache management. In [22], the authors considered a different problem than ours: replacement of locally-cached data items with new ones. As the authors claimed, the significance of this functionality stemmed from the fact that data queried in real applications were not random, but instead exhibited locality characteristics. Therefore, the design of efficient replacement policies, given an underlying caching mechanism, was addressed. In [23], although the authors considered delay aspects and a realistic industrial IoT model (based on WirelessHART), their main objective was to bound the worst-case delay in the network. Furthermore, they did not exploit the potential presence of proxy nodes, and consequently, they stuck to the traditional, centralized industrial IoT setting. In [24], the authors considered a multi-hop network organized into clusters and provided a routing algorithm and cluster partitioning. Our DML concepts and algorithm can work on top of this approach (and of any clustering approach), for example by allocating the role of proxies to cluster heads. In fact, clustering and our solution address two different problems. In [25], the authors considered industrial networks with a fixed number of already deployed edge devices that acted as proxies and focused on the maximization of the network lifetime. They considered as lifetime the time point until the first node in the network died. As a result, in this paper, they assumed a different model, different optimization problem and different performance targets. Finally, a relevant application domain for distributed data management was also the workshop networks in smart factories [26,27], in which a large amounts of data was transmitted, bringing big challenges to data transfer capability and energy usage efficiency.

Motivating examples: We also present two indicative application areas where the DML and the relevant algorithm can provide additional value. In [28], the authors presented a typical situation in an oil refinery where miles of piping were equipped with hundreds and thousands of temperature, pressure, level and corrosion sensors, which were deployed in a large geographical area. Those sensor motes not only performed industrial condition monitoring tasks, but they also used the industrially-oriented communication technology TSCH. In [29], the authors presented two large-scale deployments of hundreds of nodes; one in a semiconductor fabrication plant and another on-board an oil tanker in the North Sea. They used Mica2 and Intel Mote nodes, very similar to our sensor motes of choice. In both those applications, due to the exact fact that the sensor motes were not able to communicate directly with the controller (transmission range restrictions), the system designers naturally considered a multi-hop propagation model. The targeted decentralization of the data distribution process in those large-scale condition monitoring and predictive maintenance application areas could lead to economic benefits for the industrial operator and maintenance of some important metrics in the network at good levels, while ensuring that the end-to-end latency is acceptable, without introducing overwhelming costs in the system for the purchase of expensive equipment.

## 3. System Modeling

The network: We consider networks of industrial IoT devices that usually consist of sensor motes, actuators and controller devices. We model those devices as a set of $S=\{s_{1},s_{2},\ldots ,s_{n}\}$ nodes, with a total number of |S|=n nodes, deployed in an area of interest A . The central network controller *C* is set as the first device in the network, $C=s_{1}$ . The communication range $r_{u}$ of a node *u* varies according to the requirements of the underlying routing protocol and the constraints of the technological implementation. Nodes u,v∈S are able to communicate with each other if $r_{u},r_{v}\geq \epsilon \left(u,v\right)$ , where ϵ(u,v) is the Euclidean distance between *u* and *v*.

We assume that the controller *C* is able to maintain centralized network knowledge. This is usual in industrial applications, in which the locations of the nodes are known, traffic flows are deterministic and communication patterns are established a priori. We assume that *C* knows all the shortest paths in the network and comes with an n×n matrix D , where $D_{u,v}$ is the length of the shortest path between nodes *u* and *v* (the offline shortest path computation between two nodes is a classic problem in graph theory and can be solved polynomially, using Dijkstra’s algorithm [30]). Note that only the control of the data management plane is centralized, while, when using the DML, the data plane itself can be distributed and cooperative, by storing the data in proxies. A proxy *p* is a node that is able to store data that can be accessed in a timely manner from the consumer nodes of the network. The set of all proxies is denoted as *P*, with p∈P⊂S . The DML ensures the effective proxy cooperation so as to achieve the optimal performance of the network according to the objective function defined next. The network controller *C* is also serving as a proxy, and thus, we have that |P|≥1 in all cases.

Data production and consumption: In typical industrial applications, like condition monitoring, sensor nodes perform monitoring tasks (producers), and in some cases, their sensor data are needed either by other sensor nodes, which could need additional data to complement their own local measurement, or by actuator nodes, which use the sensor data so as to perform an actuation (consumers). When needed, a consumer *u* can ask for data of interest using the primitives defined by the underlying routing protocol from a sensor node *v* (ideally from a proxy *p*, when using the DML). We define the set of consumers as $S_{c}\subset S$ , with $|S_{c}|=m<n$ . When a consumer *u* needs data, it requests the data via a multi-hop routing path, from the corresponding proxy *p*. When *p* receives the data request from *u*, it sends the requested data along the same routing path, starting at *p* and finishing at the consumer *u*. Note that the length of this individual data delivery path is twice the length of the path between *u* and *p*. We assume that the data generation and access processes are not synchronized. Specifically, we assume that data consumers request data at an unspecified point in time after data have been generated by data producers and transferred to the proxies.

**Figure 2:**
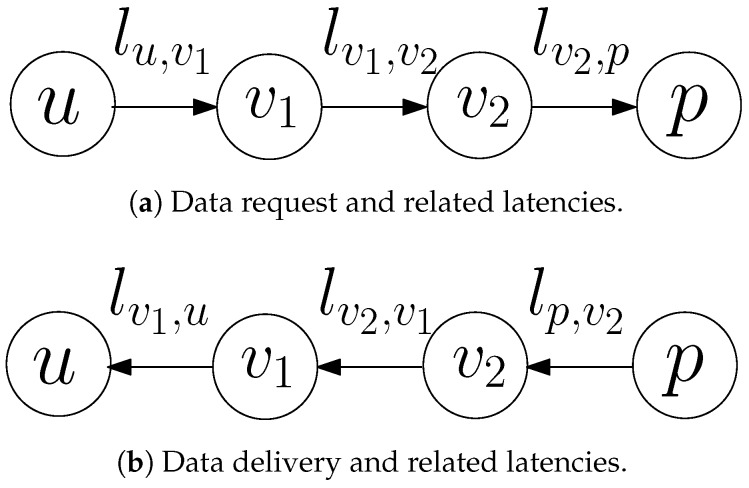
Example of data access latency. In this case, the data access latency is $L_{u,p}=l_{u,v_{1}}+l_{v_{1},v_{2}}+l_{v_{2},p}+l_{p,v_{2}}+l_{v_{2},v_{1}}+l_{v_{1},u}$ .

The latency constraint: Let $l_{u,v}$ be the single-hop data transmission latency from a node u∈S to another node v∈S . We define as access latency $L_{u,p}$ the amount of time required for the data to reach consumer *u*, after *u*’s request, when the data follow a multi-hop propagation between *u* and *p*. We denote access latency as $L_{u,p}=l_{u,v_{1}}+\ldots +l_{v_{i},p}+l_{p,v_{i}}+\ldots +l_{v_{1},u}$ . An example of the access latency composition is depicted in Figure 2. We denote as $\bar{L}$ the average access latency across all consumers as the mean value of all the latencies $L_{u,p}$ . More specifically, $\bar{L}$ can be denoted as the quantity:

(1) $$\bar{L}=\frac{\sum_{\forall u\in S_{c}}L_{u,p}}{m},$$

where *m* is the number of consumers.

Industrial applications are typically time-critical, and consequently, the industrial operator requires a maximum data access latency threshold $L_{\max}$ . This is an important constraint in the network, and the implementation of a data delivery strategy should ensure that the average multi-hop access latency does not exceed the threshold value. In other words, the following inequality should hold: $\bar{L}\leq L_{\max}$ . Note that this formulation is amenable to different purposes. If $L_{u,p}$ is the mean latency between *u* and *p*, the above inequality guarantees that the average of the mean latencies is below $L_{\max}$ . If it is the worst-case latency between *u* and *v*, the inequality provides a guarantee on the average worst-case latency. In the following, we show that the DML can be used in both cases.

## 4. The Data Management Layer

**Figure 3:**
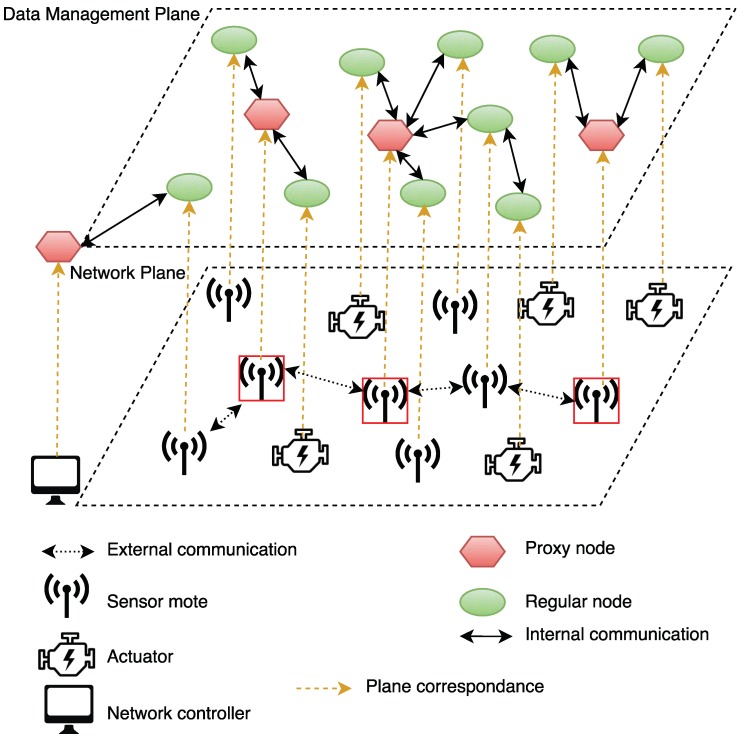
The Data Management Layer (DML): decoupling the data management plane from the network plane.

In order to manage the data distribution process and decrease the average access latency in the network, we consider a DML similar to the one defined in [17], which we recap here for the reader’s convenience. The basic function of the DML is the decoupling of the data management plane from the network plane, as shown in Figure 3. The DML provides solutions for the selection of some nodes that will act as proxies and the establishment of an efficient method for data distribution and delivery, using the proxies. More specifically, the role of the DML is to define a set P⊂S , the elements of which are the selected proxies. The number of the proxies can range from 1– n−1 . The case of one proxy is equivalent to having only the controller *C* operating as a single point of data distribution. In this case, the data distribution is functioning as in traditional industrial IoT environments.

This demarcated model of data exchanges can be formulated as a publish/subscribe (pub/sub) model [31]. In a pub/sub model, a consumer subscribes to data, i.e., denotes interest for it to the corresponding proxy, and the relevant producer publishes advertisements to the proxy. The DML assumes that the pub/sub process is regulated at the central controller *C*, which maintains knowledge on the sets of producers, consumers and requests. Based on this, *C* can find an appropriate set of proxies based on the algorithm we present next. Inside the network, the proxies are responsible for matching subscriptions with publications, i.e., they provide a rendezvous function for storing the available data according to the corresponding subscriptions. The producers do not hold references to the consumers, neither do they know how many consumers are receiving their generated data.

The selection of the proxies should be done balancing two requirements. On the one hand, the number of proxies should be sufficient to make sure each consumer finds data “close enough” to guarantee that $\bar{L}\leq L_{\max}$ . On the other hand, as the role of proxy implies a resource burden on the selected nodes, their number should be as low as possible. The proxy selection problem can thus be formulated as an integer program. More specifically, given a set *S* of nodes, a set $S_{c}\subset S$ of consumers and an access latency threshold $L_{\max}$ , the network designer should target the minimization of the number of proxies needed in the network so as to guarantee $\bar{L}\leq L_{\max}$ . We define two sets of decision variables, (a) $x_{p}=1$ , if p∈S is selected as proxy and zero otherwise, and (b) $y_{u,p}=1$ , if consumer u∈S is assigned to proxy p∈S and zero otherwise. Then, the integer program formulation is the following:

(2)Min.:∑p∈Sxp(3)S.t.:∑u∈Sc∑p∈SLu,p·yu,pm≤Lmax(4)∑p∈Syu,p=1∀u∈Sc(5)yu,p≤xp∀u∈Sc,∀p∈S(6)xp,yu,p∈{0,1}∀u∈Sc,∀p∈S

The objective function (2) minimizes the number of proxies (note that in various industrial scenarios, some nodes might be too weak to perform any other operations than generating and propagating a minimal set of data. The problem formulation that represents those scenarios is a special case of the problem formulation that we consider in this paper, with $p\in S^{\prime }$ , where $S^{\prime }\subset S$ ). Constraint (3) guarantees that $\bar{L}\leq L_{\max}$ . Constraints (4) guarantee that each node has to be assigned to one and only one proxy. Constraints (5) guarantee that nodes can be assigned only to proxies. Constraints (6) guarantee that all nodes are considered for potentially being selected as proxies and that all nodes requesting data are assigned to a proxy.

As we have already shown in [17], the proxy selection problem is computationally intractable, since it can be formulated as an integer program. This means that it is impossible to calculate in polynomial time the minimum proxies needed optimally while staying below $L_{\max}$ . Differently from [17], in this case, the formulation of the problem considers the latency of communication $L_{u,p}$ , and not an abstract number of hops. This makes it more realistic for industrial environments, but an even more difficult problem, as it becomes also infeasible to assign the real values to $L_{u,p}$ of Constraints (3). This is due to the fact that we are not able to know the exact values of the individual transmission latencies $l_{u,v}$ , before they happen. To address this issue, we introduce the ProxySelection+ algorithm (Algorithm 1), which takes into account latencies $L_{u,p}$ , instead of the number of hops. ProxySelection+ is a myopic algorithm, which does not give the optimal solution. The use of simple heuristics like the one in ProxySelection+ shows that the DML is able to outperform the traditional centralized methods, even when adopting simple methods.

ProxySelection+ sets the controller *C* as the first proxy of the network, and it gradually increases the number of proxies (counter) until it reaches a number with which the average access latency $\bar{L}$ does not violate the maximum latency threshold $L_{\max}$ . In every iteration (Lines 5–9), the exact selection of the next proxy in the network is performed using a myopic greedy addition (Lines 6–8). Each candidate node is examined, and the one whose addition to the current solution reduces the average access latency the most is added to the incumbent solution. To this end, the latency between a candidate proxy (*k* in Line 7) and a consumer (*u* in Line 7) is estimated as the length of the shortest path $D_{k,u}$ that is connecting them multiplied by the expected latency on each hop ( $l^{\left(h\right)}$ in Line 7). $l^{\left(h\right)}$ needs to be initiated through preliminary measurements. This happens through an initialization phase (Line 1) during which the network designer measures different single-hop data transmission latencies within the industrial installation and gathers a sufficiently representative dataset of $l_{u,v}$ measurements from different pairs of nodes u,v∈S across the network. By using the mean of measured latencies, we obtain a guarantee on the average mean latency. By using the highest measured value, we obtain a constraint on the average worst-case latency, implementing the guarantee explained in Section 3. The computational complexity of ProxySelection+ is polynomial with a worst case time of $O(V^{4})$ . However, this worst-case performance is very difficult to experience in practice, since in order to have $O(V^{4})$ time, all *n* nodes of the network have to be chosen as proxies; something that is highly unlikely.
**Algorithm 1:**
ProxySelection+.
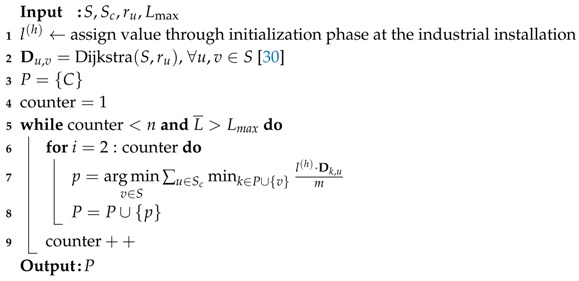


## 5. Implementation and Experimental Evaluation

### 5.1. Experimental Strategy

Strategic purpose: The strategic purpose of the experimental evaluation with real devices is to provide a realistic demonstration of how efficient data management methods can significantly improve the data access latency in industrial IoT networks, by using a limited number of proxies. The realistic approach is of paramount importance in our implementation strategy. For this reason, we follow some important steps. For the experimental implementation and evaluation, we use the Euratech testbed from the FIT IoT-LAB platform [14]. We use a network with technical specifications representative of the industrial IoT paradigm (e.g., low-power nodes, IEEE 802.15.4 radio interface, large number of devices, etc.). We carefully choose the $L_{\max}$ threshold, according to actual industrial requirements and expert groups’ recommendations. In order to have a benchmark for the performance of our method, we also implement two additional representative methods, based on routing mechanisms that are usual in current industrial IoT networks. We vary several experimental parameters so as to investigate the performance consistency of our method under different settings. Finally, we validate a simulation model based on the real-world settings, with which we can further investigate the changing parameters that are impossible or too time-consuming to investigate on the testbed.

**Figure 4:**
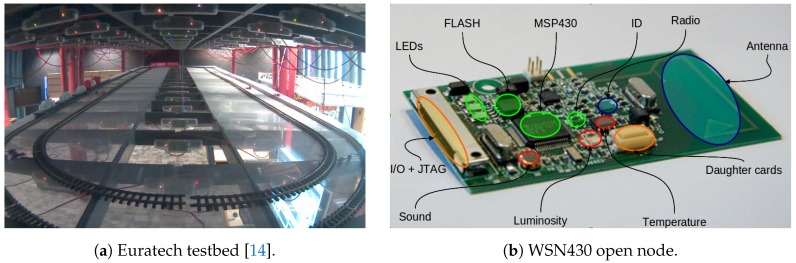
Experimental setup.

Experiment design: We use a total number of n=95 nodes in the Euratech testbed, which form a 2D horizontal grid, as shown in Figure 4a. Occasionally, during the experiments, there are some dead nodes, that is nodes that have run out of available power and are not able to function. This occasional unavailability of a subset of nodes renders the experiment even more realistic, since dead node situations frequently occur in real industrial IoT networks. The nodes that were used in the experiments are WSN430 open nodes, the design of which is displayed in Figure 4b. The WSN430 open node is a mote based on a low-power MSP430-based platform, with a set of standard sensors and an IEEE 802.15.4 radio interface at 2.4 GHz, using a CC2420 antenna [32], which can support, e.g., WirelessHART settings, typical of industrial communications. We used the TinyOS configuration for CC2420, which uses a MAC protocol that is compatible with 802.15.4 and is in principle a CSMA/CA scheme. We programmed and operated the nodes under TinyOS 2.1.1, a reference operating system for sensor nodes.

**Figure 5:**
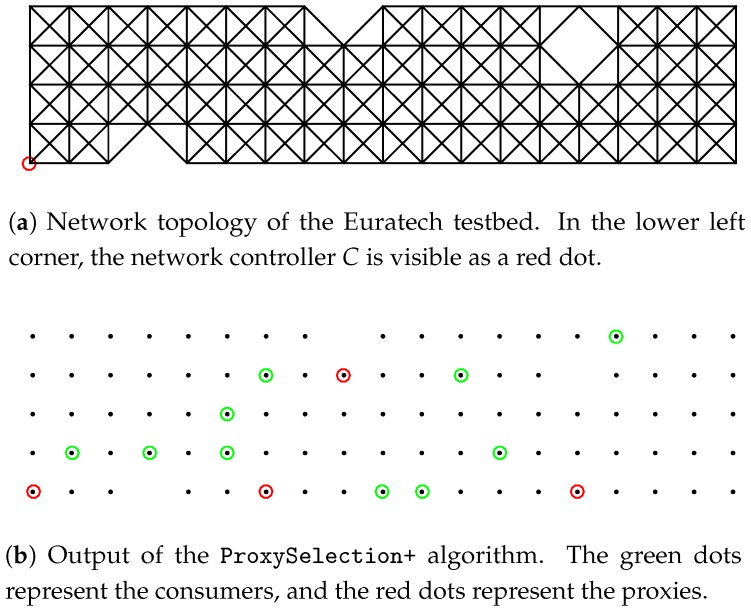
Euratech topology and ProxySelection+ output.

Since the testbed nodes are placed a short distance from each other, we adjust their transmission range, so as to obtain a realistic multi-hop topology. We configured the antenna TX power such that, according to the CC2420 antenna datasheet [32] and the measurements provided in [33], the transmission range is about 3 m. However, given that this value has been measured in ideal conditions, without taking into account external factors such as obstacles and interference, we program every node u∈S to consider as a neighbor every other node v∈S with ϵ(u,v)≤1 m. Given this configuration, we obtain the topology that is depicted in Figure 5a. Note that the three “gaps” in the topology are a result of the dead nodes of the deployment, which are unable to communicate with other nodes. We set the percentage of requesting nodes to m=0.1·|S| , selected uniformly at random from *S*, and we set $C=s_{1}$ as the central network controller, which corresponds to the node with node_id =1 in the Euratech testbed (lower left node in Figure 5a).

Setting the $L_{\max}$ threshold: In order to perform the experiments in the most realistic way, it is important that the $L_{\max}$ value be aligned with the official communication requirements of future network-based communication solutions for Industry 4.0, for the targeted industrial applications. Both the WG1 of Plattform Industrie 4.0 (reference architectures, standards and norms) [34] and the Expert Committee 7.2 of ITG (radio systems) set the latency requirements for condition monitoring applications to 100 ms. However, in order to provide a complete and diverse set of results, we also measure the performance of our method for different values of $L_{\max}$ .

Performance benchmarks: In order to measure the performance of the DML with respect to traditional industrial IoT alternatives, we implement two additional data delivery strategies. The first method is the most traditional data delivery strategy in current industrial IoT environments and imposes that all data requests and data deliveries are being routed through the controller *C*. More specifically, the request is routed from consumer *u* to *C* and then from *C* to producer *v*. At the next step, the data are routed from *v* again to *C* and then from *C* to *u*. We call this mode of operation non-storing mode, and it is obvious that it is completely centralized and not cooperative. Note that this would be the simplest data management approach that can be implemented in relevant routing mechanisms like RPL (IPv6 Routing Protocol for Low-Power and Lossy Networks [35]), where intermediate nodes are not allowed to cache data (thus, the RPL terminology non-storing mode).

The second method is another, less commonly used in industrial IoT settings, but nevertheless useful alternative. It imposes that all data requests and data deliveries are being routed through the Lowest Common Ancestor (LCA) of the routing tree, routed at the controller *C*. The LCA of two nodes *u* and *v* in the routing tree is the lowest (i.e., deepest) node that has both *u* and *v* as descendants. We call this mode of operation storing mode, because the LCAs should store additional information about their descendants, and it is obvious that it is a distributed alternative. Again, this is the simplest method that one would implement with routing mechanisms like RPL in storing mode, i.e., when intermediate nodes between communication endpoints are allowed to cache content. Storing mode thus provides a distributed method.

We made those choices after careful consideration of the current realistic industrial networking status-quo. The selected protocols are standardized components of a reference and well-established communications and data management stack. In fact, they are considered the state-of-the-art, for current and future wireless industrial applications, as discussed extensively in [15,16]. More specifically, the stack is presented in detail in [15] and is considered as the de facto standard for future industrial networks. In the following, for convenience, we use the term “special nodes” when we refer to the network controller, the proxies or the LCAs.

### 5.2. Experimental Results

Running the ProxySelection+ algorithm: We ran the initialization phase of ProxySelection+, so as to assign values to $l^{\left(h\right)}$ , by measuring times that are needed for the data exchange of a sensor measurement from a sensor node to another. We measured the time needed for the sensor reading to be sent and received from one node to another. This latency includes time spent in the back-off phase (which cannot be predicted), time spent in sending the signal over the radio, and time spent during the propagation. We consider the propagation latency negligible, since radio waves are traveling very fast and we are not able to measure the time elapsed using the nodes’ timers. In order to obtain reliable results, we repeated the propagation measurements for different pairs of transmitting and receiving nodes of the Euratech testbed, 30 times for each pair. We concluded with the measurements that are shown in the Table 1 (highest, lowest, mean value and standard deviation), after measuring the relevant times using WSN430 with CC2420 and TinyOS. We can see that the latency values of data propagation from one node to another significantly vary. While the lowest latency could be 13 ms, the highest propagation latency $l^{\left(\max\right)}$ was 23 ms. The mean latency $l^{\left(\mathrm{mean}\right)}$ of the values collected from the repetition of this experiment was 17.4 ms (other sources of latency related, e.g., to computation at the receivers have been found to be in the order of μ s and, therefore, are neglected. Furthermore, the measuring methodology we used does not depend on the specific conditions under which these measures are taken). After running ProxySelection+ with $L_{\max}=100$ ms, m=0.1·|S| and $l^{\left(h\right)}=l^{\left(\mathrm{mean}\right)}$ , we get the proxy placement that is depicted in Figure 5b. We can easily see that ProxySelection+ is balancing the proxy allocation in the network, so as to guarantee a small data access latency to all the requesting nodes.

**Figure 6:**
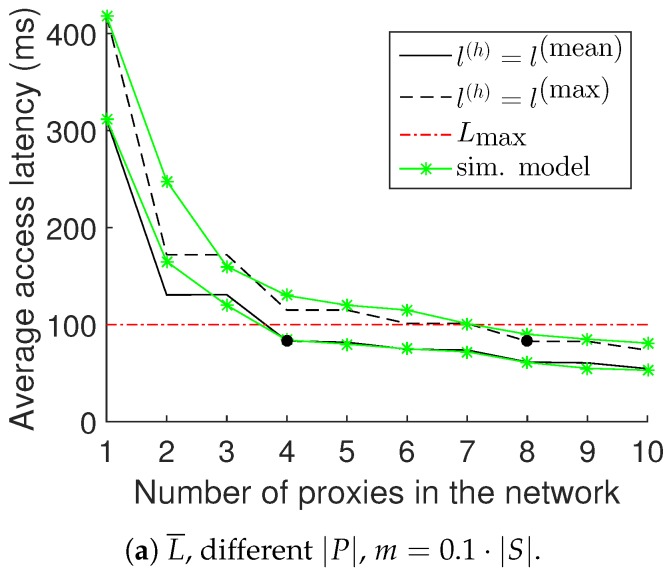

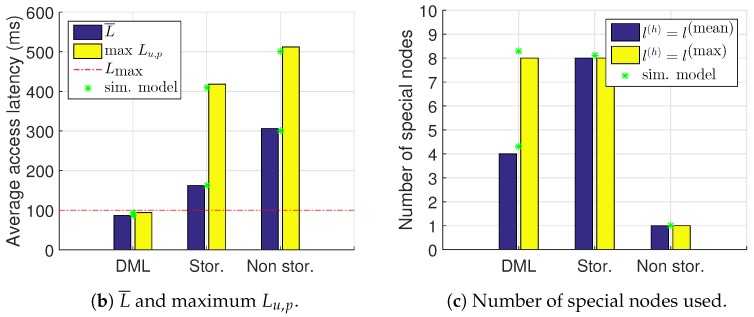
Experimental results in the IoT-LAB Euratech testbed. The validation of the simulation model is displayed in green.

Increasing and decreasing the number of proxies: Figure 6a displays the average access latency $\bar{L}$ for different numbers of proxies in the network. In order to obtain this plot, we run ProxySelection+, and we gradually add and remove proxies, so as to investigate the effect of changing the number of proxies on $\bar{L}$ . In the case where we set $l^{\left(h\right)}=l^{\left(\mathrm{mean}\right)}$ , the DML ensures that $\bar{L}$ will not surpass $L_{\max}$ , by assigning four proxies in selected positions. If we further decrease the number of proxies, we have that $\bar{L}>L_{\max}$ , and the latency constraint is not met. At the leftmost point of the plot, we can see the latency achieved when using only one proxy (the controller *C*, or in other words, when the DML functionalities are absent), which is much higher than when employing additional proxies. When we replace the value of $l^{\left(h\right)}$ with $l^{\left(h\right)}=l^{\left(\max\right)}$ and we re-run the algorithm, we observe similar behavior in the performance, but in this case, with eight selected proxies.

$\bar{L}$ achieved: Figure 6b displays the results on the average access latency for the three alternative methods. The yellow bar for the DML method is the $\bar{L}$ value when we consider the worst case of $l^{\left(h\right)}=l^{\left(\max\right)}$ . This is an important point to make, as the figure shows that, by adapting the number of proxies, DML is able to always guarantee the constraint, irrespective of whether $l^{\left(h\right)}$ is formulated as an average of mean latencies or as an average of worst-case latencies. We can see that the efficient management of proxies provided by the DML results in a better performance compared to the other two alternatives. This fact is explained by the nature of ProxySelection+, which receives as input the $L_{\max}$ .

Number of proxies used: We compare the three methods with respect to the number of special nodes that they use. The DML is using proxies; the non storing mode is using the controller *C*; and the storing mode is using LCAs. The use of special nodes is wasteful of resources. For example, the proxies store the data requested and the correspondence of producers and consumers, and the LCAs hold routing information about their descendants. In Figure 6c, we can see that the DML is performing really well compared to the storing mode and uses less special nodes. Of course, the non-storing mode is using just one special node, but this has a severe impact on the latency achieved, as shown in Figure 6b. Even when the DML uses more proxies to guarantee worst-case latencies, their number is comparable to the case of storing mode. However, the DML drastically reduces the latency in this case, thus achieving a much more efficient use of proxies.

## 6. Large-Scale Simulations

The testbed environment gives us an important ability to test the methods on real conditions and derive useful indications. However, at the same time, it does not allow us to perform larger scale, or variable experiments, easily and fast. For this reason, we developed a simulation model based on the system modeling presented in Section 3. The simulation environment we use is MATLAB. We verify that the simulations are meaningful via validation, by comparing the results obtained with the simulation model to those of the testbed experiments, and then, we extend our performance evaluation through simulations.

### 6.1. Validation of the Simulation Model and Simulation Settings

We constructed, in simulation, instances similar to the one that was tested in the Euratech testbed. The results obtained are displayed with green color in Figure 6. It is clear that the results obtained by the simulation model are very similar to the results obtained during the real experiment, and therefore, we can extract reliable conclusions from the simulation environment.

**Figure 7:**
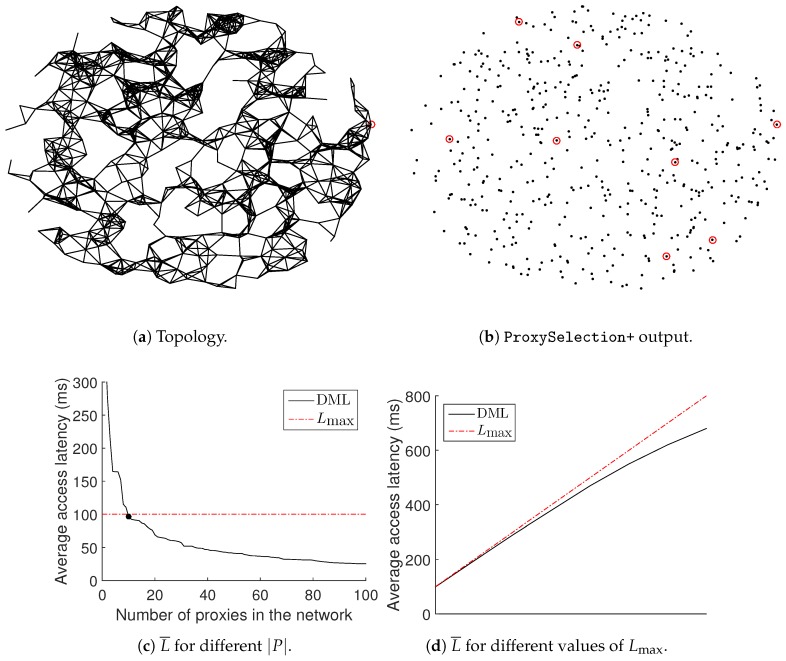
Network with n=500 , m=0.4·|S| .

Figure 7a displays a typical network deployment of 500 nodes, with the corresponding wireless links and with the controller *C* lying on the far right edge of the network, depicted as a red circle. Figure 7b displays the locations of the final set *P* of proxies depicted as red circles after running ProxySelection+. The spatial display of Figure 7b shows that the final selection results in a balanced proxy selection, ensuring that even isolated nodes, which are located near sparse areas of the network, also have access to a proxy.

In the simulations, we focus on showcasing different aspects of the data management and distribution process. We construct larger and different deployments and topologies than the ones of the Euratech testbed; we investigate different values of $L_{\max}$ ; we consider diverse percentages of requesting nodes; and we also measure the energy consumption. The deployment area A is set to be circular, and the nodes are deployed uniformly at random. We construct networks of different numbers of nodes, inserting the additional nodes in the same network area and at the same time decreasing the communication range $r_{u}$ appropriately, so as to maintain a single strongly-connected component at all times. An example of a generated network of 500 nodes is depicted in Figure 7a. In the following, we present results where $l^{\left(h\right)}$ is measured as the mean of latencies. Figure 7c shows the value of $\bar{L}$ obtained in the case of Figure 7a, qualitatively confirming the results shown in Figure 6a.

### 6.2. Simulation Results

Different values of $L_{\max}$ : We tested the performance of the DML for different values of $L_{\max}$ , in networks of 500 nodes, with m=0.4·|S| . The results are shown in Figure 7d. The red points represent the values for the maximum latency threshold $L_{\max}$ provided by the industrial operator. The average access latency achieved by the DML is always below the threshold, due to the provisioning of the ProxySelection+ algorithm. In fact, we can see that the more the value of $L_{\max}$ is increased, the larger the difference between $\bar{L}$ and $L_{\max}$ becomes. This happens because for higher $L_{\max}$ values, the latency constraint is more relaxed, and lower $\bar{L}$ can be achieved more easily.

**Figure 8:**
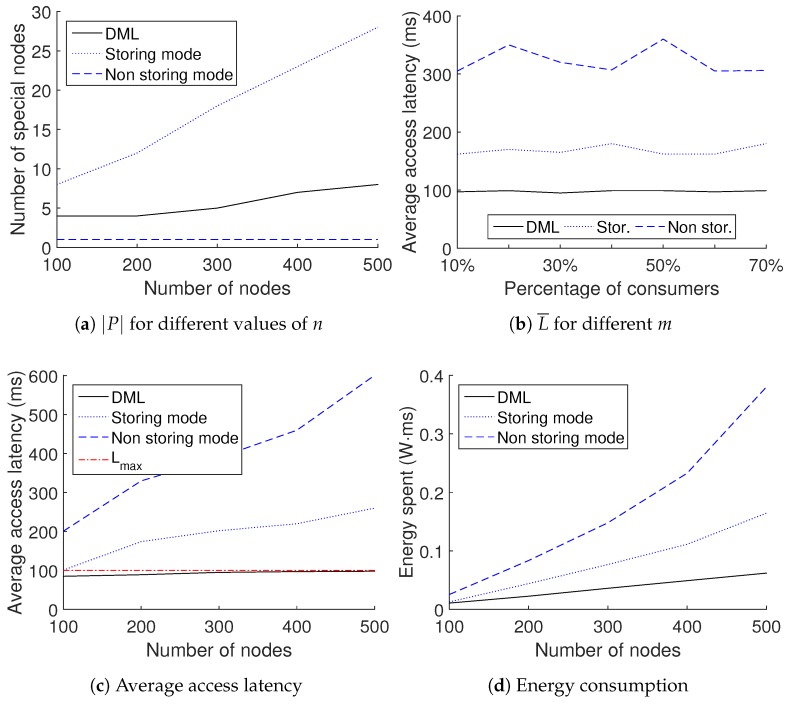
Comparison of the three methods.

Number of proxies used: We compare the three methods with respect to the number of special nodes that they use. As usual, the DML is using proxies; the non-storing mode is using the controller *C*; and the storing mode is using LCAs. As we mentioned earlier, the use of special nodes is wasteful of resources. In Figure 8a, we can see that the DML is performing really well compared to the storing mode and uses much less special nodes. Of course, the non-storing mode is using just one special node for any network size, but this has a severe impact on the latency achieved.

Different percentages of requesting nodes: Another possible factor that could affect the $\bar{L}$ achieved is the percentage of consumers. In Figure 8b, we can see that $\bar{L}$ remains constant for any percentage of requesting nodes, in all three alternatives. This shows that DML is able to automatically adapt the number of proxies, so as to guarantee the latency constraints irrespective of the number of consumers.

Average latency achieved: Figure 8c displays the results on the average access latency for the three alternatives, for different numbers of nodes in the network. We can see that the efficient management of proxies provided by the DML results in a better performance compared to the other two alternatives. $\bar{L}$ achieved by the DML respects the latency constraint and always remains lower than $L_{\max}$ (red line).

Energy consumption: Another aspect that we can easily evaluate in the simulation is the energy cost in terms of communication, related to data access. We evaluate this as the cost of transmissions required to serve consumers’ requests. In order to obtain the desired results in units of energy, we transform the dBm units provided in the CC2420 datasheet [32] to mW and we multiply with the time that each node of the network is operational. Figure 8d displays the energy consumption in the entire network for the three alternatives, for different numbers of nodes. The energy consumption for communication is lower in the case of the DML because low latency comes with less transmissions in the network, resulting in fewer energy demands.

## 7. Conclusions and Future Work

In this paper, we efficiently regulate the data distribution in industrial IoT networks using proxies. Given a set of data, the set of consumer nodes and the maximum access latency that consumers can tolerate, we consider a method for identifying a limited set of proxies in the network where data are cached. We implement the method and evaluate its performance using the IoT-LAB testbed. Additionally, we validate a simulation model and use it for performance evaluation in larger scales and more general topologies. We demonstrate that the proposed method guarantees average access latency below the given threshold and outperforms traditional centralized and even distributed approaches. The next step is to take into account limited bandwidth in the network, which can lead to congestive collapse, when incoming traffic exceeds outgoing bandwidth.

## Figures and Tables

**Table 1:** Measured send/receive latency.

Type of Measured Latency	Notation	Value (ms)	σ
**Highest latency reported**	$l^{\left(\max\right)}$	23	
**Mean latency**	$l^{\left(\mathrm{mean}\right)}$	17.4	3.2
**Lowest latency reported**	-	13	
